# Detection of *Leishmania donovani* using ITS1-RFLP from positive and negative smear samples among clinically reported patients visiting University of Gondar Comprehensive Specialized Hospital

**DOI:** 10.1186/s12879-022-07930-1

**Published:** 2022-12-29

**Authors:** Umer Ahmed Usmael, Nega Berhane Tesema, Selfu Girma, Desalegn Adane Kendie, Musin Kelel Abas

**Affiliations:** 1 Departments of Medical Biotechnology, University of Gondar, Ethiopia and Addis Ababa Science and Technology University, Addis Ababa, Ethiopia; 2 Department of Medical Biotechnology, University of Gondar, Gondar, Ethiopia; 3 Armauer Hansen Research Institute (AHRI), Addis Ababa, Ethiopia; 4 Leishmania Research and Treatment Centre, University of Gondar Comprehensive Specialized Hospital, Gondar, Ethiopia; 5 Departments of Biotechnology, Addis Ababa Science and Technology University, Addis Ababa, Ethiopia

**Keywords:** Diagnosis, *Leishmania donovani*, ITS1, PCR-RLFP, Smear-negative, Smear-positive

## Abstract

**Background:**

Visceral leishmaniasis is caused by the *Leishmania donovani* species complex that can spread to internal organs and leading to death if not treated on time. Diagnosis of leishmaniasis is based on clinical signs and symptoms, microscopy, serological and molecular techniques. Because of a broad spectrum of diverse clinical manifestations and similarities of the responses to different species, identification to the species level is often difficult for the proper patient treatment and management. Therefore, the objective of this study was to evaluate the PCR- RFLP assay of the ITS1 region for identification of *L. donovani* species from clinical smear slide patient samples.

**Method:**

DNA extraction was performed on a total of 90 smear slide samples using phenol—chloroform method. The PCR detection limit was determined by *L. donovani* reference strain DNA. The ITS1 region was amplified at 320 bp using LITSR/L5.8S genus specific primers and then the ITS1-PCR products were subjected to RFLP assay for confirmation of *L. donovani* species using *Hae*III restriction enzyme.

**Results:**

Of the total samples ITS1-PCR revealed the true positive, false positive, true negative, and false negative results of 42 (46.7%), 6 (6.7%), 37 (41.1%) and 5 (5.6%), respectively. Considering microscopy as the gold standard, the sensitivity, specificity, positive predictive values, and negative predictive values of the ITS1- PCR technique was 89.4%, 86.0%, 87.5%, and 88.1% respectively. All ITS1-PCR positive clinical samples were confirmed as *L. donovani* species by PCR–RFLP patterns.

**Conclusion:**

In conclusion, the ITS1- RFLP method is highly sensitive and more specific for identification of *L. donovani* species in the smear negative clinical samples of visceral leishmaniasis patients. There is also significant association and degree of agreement between the two methods. For direct identification of *L. donovani* species from clinical samples, irrespective of genus and species level, PCR–RFLP is more recommendable than a microscope.

**Supplementary Information:**

The online version contains supplementary material available at 10.1186/s12879-022-07930-1.

## Background

Leishmaniasis is a vector-borne neglected parasitic tropical disease caused by protozoa of the genus *Leishmania* [1]. According to the World Tropical Diseases Research Center, it is classified as one of the top three parasitic diseases along with African trypanosomiasis and dengue fever [2]. Infections with *Leishmania* are endemic in wide parts of the tropics, subtropics, and Mediterranean basins, affecting over 98 countries. There are also millions of new cases reported annually, of which the highest new cases are cutaneous leishmaniasis (CL) [3–5]. Among all newly reported *Leishmania* cases, visceral leishmaniasis (VL) caused by the *L. donovani* species complex are the leading cause of mortality because of spreading to the internal organs [4, 6, 7]. If not treated on time it is the most lethal type of Leishmaniasis with an almost 100% mortality rate [4].

East Africa is the second leading VL focus after the Indian subcontinent, contributes to the worldwide burden with 30,000–40,000 new cases per year, of which Ethiopia, South Sudan, and Sudan contributing the largest proportion of the cases [8]. There are two main populations that serve as the primary sources of *L. donovani* strains, one of which contains strains from northern Ethiopia and Sudan and the other of which contains strains from southern Ethiopia and Kenya [9]. The two primary sand fly vectors, *Phlebotomus orientalis* and *Phlebotomus martini*, are present in these areas, although other vectors have been implicated. *Phlebotomus orientalis* is the dominating vector in northern Ethiopia and Sudan, while *Phlebotomus martini* is the main vector in southern Ethiopia and Kenya [10].

Over 3.2 million Ethiopians are at risk of infection, and the country perceives 400–700 new cases of VL or kala-azar each year [11, 12]. The Segen-Woito valleys, lower Omo river plains, Lake Abaya area in the southwest, and Metema-Humera low land (500–700 m above sea level) in the northwest, which account for about 60% of the cases are the known VL endemic foci [8, 13]. Visceral leishmaniasis has recently expanded to the highlands of the LiboKemkem area (South of Gondar), killing hundreds of peoples [8].

Current diagnostic methods of *Leishmania* parasite for all species are primarily based on clinical signs and symptoms and serology. Clinical signs and symptoms cannot simply be used for the identification of *Leishmania* species due to the fact that a vast differential make a diverse clinical manifestation [1, 5, 14]. Because of their high sensitivity, specificity and ability to identify Leishmaniasis in a wide spectrum of clinical samples as well as for parasite characterization, molecular methods have become increasingly important [1].

*Leishmania* infections in humans can be detected and diagnosed using a variety of PCR protocols, including the conventional single-step PCR (CPCR), quantitative real-time PCR (qPCR), nested PCR (NPCR), PCR-Restriction Fragment Length Polymorphism (RFLP) analysis and nucleic acid sequence-based amplification (NASBA) [15]. Multi-copy sequences are chosen as targets for amplification in order to achieve high sensitivity, including kinetoplast DNA (kDNA), telomeric sequences, the gp63 gene locus, heat shock protein 70 (hsp70), cysteine protease B (cpb), cytochrome b (cyt b), the miniexon (spliced leader) gene repeat, the beta-tubulin gene region, microsatellite DNA [16–18]. The kDNA PCR was shown to have > 90% sensitivity and 100% specificity in clinical samples. Although these PCR techniques are genus-specific, they cannot distinguish between various *Leishmania* species [19].

Ribosomal internal transcribed spacers have polymorphic regions that can be employed in RFLP assays, which are used to identify and distinguish between the infecting *Leishmania* species as well as highly conserved areas that allow for their use in PCR for diagnostic purposes [20]. Restriction fragment length polymorphism analysis of PCR-amplified sequences of multi-copy genes such as ITS has been widely used to identify all types of *Leishmania* species and subspecies levels [14, 19]. There is limited research in Ethiopia in this area, especially in the *Leishmania donovani* species complex using PCR- RFLP method. The objective of this study was to evaluate the PCR–RFLP assay of the ITS1 region for direct identification of *Leishmania donovani* species from clinically reported as smear positive and negative slide samples. Therefore, identification to species level using RFLP is important for patient management and future species-specific therapeutic approaches.

## Materials and methods

### Study area and period

The study was conducted from April 2021 to September 2021 at University of Gondar Comprehensive Specialized Hospital in northern Ethiopia. In the study area there is two diagnostic and treatment center for visceral leishmaniasis with the support of the Drugs for Neglected Diseases Initiative (DNDi), one from Abdurafi health center which was stopped giving services due to current conflict in the country and the other is from University of Gondar, Comprehensive Specialized Hospital. The routinely collected data and clinical samples were obtained from the main *Leishmania* Treatment and Research Centre (LRTC) Gondar, in Northern Ethiopia. LRTC is a clinical trial site for VL using parasitological and immunological techniques where molecular tests are rarely performed. It also provides routine treatment for VL and CL patients.

### Sample size determination

The sample size required for the study was calculated according to the following standard formulas [21]

$${\text{TP}} + {\text{FN}} = \frac{{{\text{Z}}^{2} \times {\text{SN}}\left( {1 - {\text{SN}}} \right)}}{{{\text{W}}^{2} }}$$

$${\text{TN}} + {\text{FP}} = \frac{{{\text{Z}}^{2} \times {\text{SP}}\left( {1 - {\text{SP}}} \right)}}{{{\text{W}}^{2} }}$$

where TP: true positive, FN: false negative, SN: lowest acceptable sensitivity of diagnostic tests used; SP: specificity of diagnostic tests.

According to previously published data, the ITS1-PCR method was 97.6% sensitivity and 100% specificity for diagnosis [20]. Therefore, SN and SP were taken as 98% and 100% respectively for sample size calculation, Z: normal distribution value of particular confidence interval (i.e. 95%, Z = 1.96), P: leishmaniasis prevalence within the suspected group of patients. According to a recent study, the most important prevalence of VL endemic areas in Ethiopia was found in the northwest (Metema-Humera low land) 500–700 m above sea level, which accounts for approximately 60% of the cases [8]. Therefore the prevalence was taken as about 60.0% for calculation, W: represents accuracy; at 5.0% sensitivity and specificity of the confidence interval, were the standard value determined according to sample size calculation methods and N: sample size. Therefore, the total number of sample size (Ntot) used in the study was 90. N-Required for sensitivity and N-required for specificity can be calculated through the following equations:

$${\text{SN}} = \frac{{\left( {1.96} \right)^{2} \times 0.98\left( {1 - 0.98} \right)}}{0.0025} = 30.1$$

$${\text{SP}} = \frac{{\left( {1.96} \right)^{2} \times 0.01\left( {1 - 0.01} \right)}}{0.0025} = 15.21$$

$${\text{N - Required}}{\kern 1pt} {\text{for}}{\kern 1pt} {\text{sensitivity}} = \frac{{{\text{TP}} + {\text{FN}}}}{{\text{P}}} = 50.2 \sim 51$$

$${\text{N - Required}}{\kern 1pt} {\text{for}}{\kern 1pt} {\text{specificity}} = \frac{{{\text{TN}} + {\text{FP}}}}{{1 - {\text{P}}}} = 38.03 \sim 39$$

### Study design and population

A retrospective data were taken from patients attending LRTC between November 2019 and April 2021 with respective slide smear clinical samples from bone marrow and spleen aspirations, then the slides were used for ITS1-PCR (LITSR/L5.8S) test and the positive clinical samples were further confirmed by PCR–RFLP assay.

### Reference strains

The reference strain of *L. donovani* (Sidon/LGR/2), DNA samples provided by LRTC, was used as PCR positive controls.

### Sample preparation and DNA extraction

Primarily, a total 90 (47 positive and 43 negative) smear samples of bone marrow (20) and spleen (70) aspirate were obtained from LRTC and brought to the department of Biotechnology Molecular Biology laboratory University of Gondar, then stored at 4 °C until used.

Two hundred microliter (200 μl) of digestion buffer (50 mM Tris HCl, 1 mM EDTA and 1% Tween-20, PH, 8.0) was added onto the slide using a pipette to remove the bone marrow or spleen aspirates of smear material from the slides. After approximately 30 s, the same pipette tip was used to transfer it back into a pre labeled 1.5 ml eppendorf tube. A sterile blade was used to scrap and transfer any visible remaining smear residue into the eppendorf tube [22].

DNA extraction was performed according to Poljak and Barlic [23], with some modifications. Briefly, the scrapped material was re-suspended in a 200 μl digestion buffer containing 20 μl of proteinase K and then incubated at 65 °C for 1 h in a water bath. After the digestion was completed, 200 μl of phenol: chloroform (25:25) saturated with 10 mM Tris–HCL (pH 8.0) was added to each tube and shaken vigorously for 1 min followed by centrifugation (BK-THR20K) at 4 °C with 8000 rpm for 10 min. Then a supernatant containing DNA was transferred into another tube. The DNA was precipitated with 500 μl of cold absolute ethanol at − 20 °C for 40 min. After centrifugation for 5 min, the supernatant was discarded; the DNA pellet was washed twice with 500 μl of 70% cold ethanol, and centrifuged at 8000 rpm for 5 min. Finally the DNA pellet was air-dried and resuspended in 50 μl of 1 × TE buffer. The DNA concentration was determined by nano-drop (Optizen nano Q) spectrophotometer and then qualitatively confirmed by preparing 1% agarose (Pronadisa Micro & Molecular biology) containing 3 μl of 0.5 μg/ml ethidium bromide. Then 12 μl aliquots of genomic DNA, with a combination of 3 μl of loading dye were loaded into the agarose gel along with a 100-bp DNA ladder. Then visualized using gel documentation system UV transilluminator.

### PCR amplification of ITS1 region

For the quality assurance of the new test, negative control was run parallel to the positive control, including PCR reactions containing primers without template DNA. This negative control further enhanced the quality of the test by excluding any nonspecific amplification, contaminations of reagents used during DNA extraction and PCR. The lowest detection limit of the ITS1-PCR technique was tested using a standard series of a positive control with a known concentration. Tenfold dilution was prepared for the positive control which contained 100 ng/μl to 1 femtogram (fg) of DNA and they were amplified using the PCR-ITS1 assay [19].

ITS1 was amplified using forward primer LITSR (5′-CTGGATCATTTTCCGATG-3′) and reverse primer L5.8S (5′-TGATACCACTTATCGCACTT-3′) specific to the ribosomal ITS1 region that occurs between the genes encoding the small subunit (18S) ribosomal RNA and 5.8S ribosomal RNA. A 25 μl reaction mix was prepared in a PCR tube by adding 0.5 μl of 10 pmol forward primer, 0.5 μl of 10 pmol reverse primers, 12.5 μl of One*Taq*^®^ 2X mM Master mix with Standard Buffer (New England, Biolab Inc), 9.5 μl of nuclease-free water and 2 μl of template DNA. The PCR (FTC41H2D) program comprised 5 min at 95 °C for 1 cycle, followed by 35 cycles starting at 95 °C for 30 s, 53 °C for 30 s, and 72 °C for 1 min, a final elongation step at 72 °C for 5 min and final hold at 4 °C. A 2% agarose was prepared by adding 50 ml of 1 × TAE buffer and boiled using a microwave oven (WD800D-320E), then after cooled for a few mint 3 μl of 0.5 μg/ml ethidium bromide was added. Twelve microliter (12 μl) aliquots of ITS1 PCR product, with a combination of 3 μl of loading dye were loaded into the agarose gel along with a 100-bp DNA ladder. The samples were run on the agarose gel for 1–1.5 h at 80 A and 100 V, and then visualized by using gel documentation system UV transilluminator (JY045). Positive samples yield a PCR product of 320 bp amplicons [24–26].

### PCR–RFLP

The positive ITS1-PCR products were subjected to RFLP assay using BsuRI (*Hae*III) restriction endonuclease enzyme (Thermo scientific) for identification of *Leishmania* species as previously described [5, 27, 28]. Briefly, volumes of 30 µl consisting of 15 µl of nuclease-free water, 2 µl of 10 × restriction buffer, 1 µl of BsuRI (*Hae*III) 10u/µl restriction enzyme and 12 µl of PCR products was incubated at 37 ^0^C for 2 h. Fifteen micro litters of restriction products were loaded on 2.5% agarose for electrophoresis analysis. The gel was stained with 3 μl of 0.5 μg/ml ethidium bromide and the images were observed using a UV transilluminator. Finally, the PCR- RFLP assay was carried out for the confirmation of *Leishmania donovani* species, and its subsequent digestion with the restriction enzyme *Hae*III revealed three bands for *L. donovani* (50, 80, and 190 bp) as previously described by Kumar et al. [29].

### Data analysis

Data analysis was performed to determine the sensitivity, specificity, positive and negative predictive values by using cross tabulation. The Kappa (k) values and Chi square were calculated to measure the degree of agreement and an association between Microscopy and PCR- RFLP methods respectively at 95% confidence intervals using SPSS version 20. Kappa values express the agreement beyond change and a k value of 0.21–0.60 represents a fair-to-moderate agreement, a k value of 0.60–0.80 represents a substantial agreement, and a k value of > 0.80 represents almost perfect agreement. A P value < 0.05 was considered as statistically significant [30].

## Results

### VL detection from slide smears

**Fig. 1:**
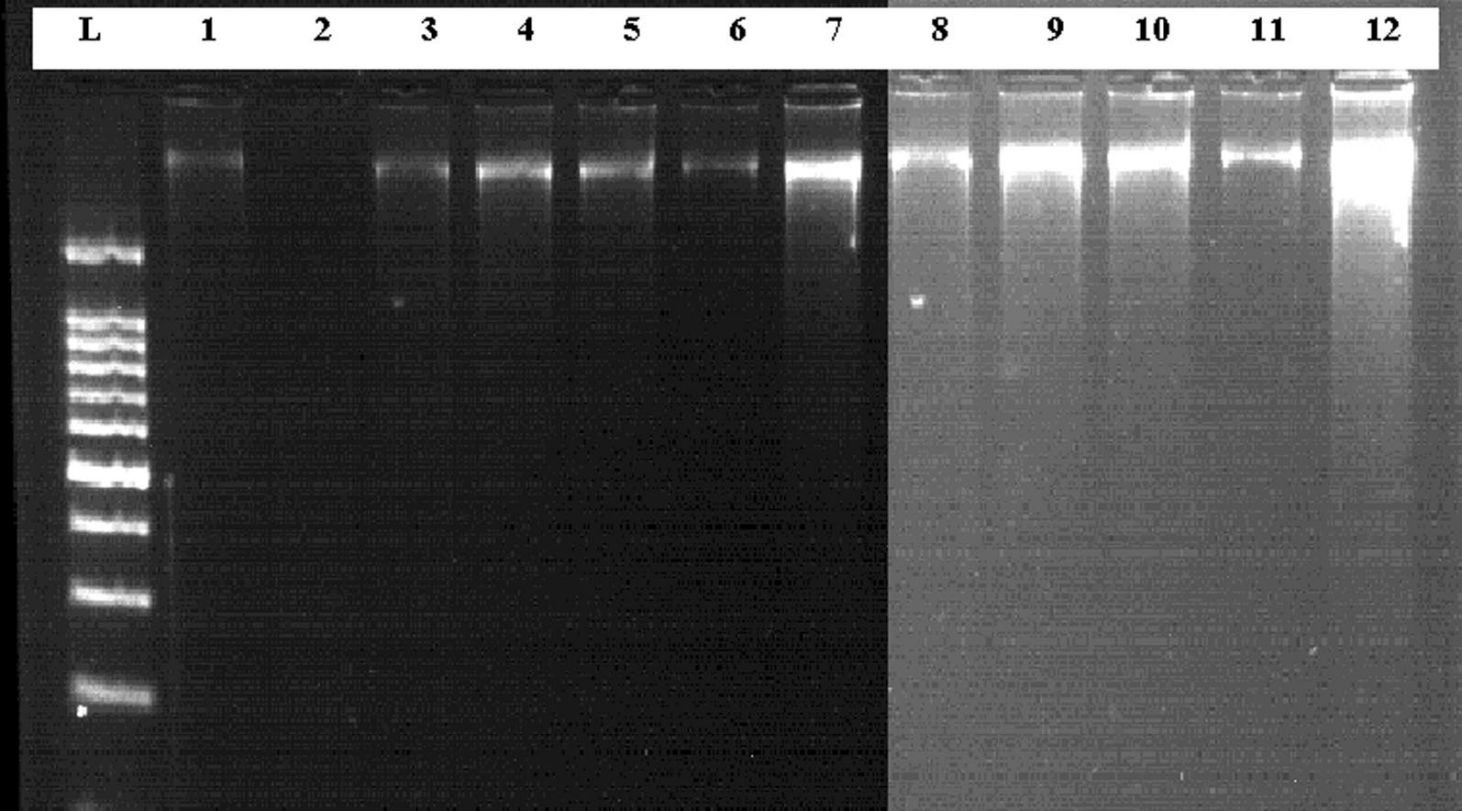
A representative of genomic DNA extracted from Patients smear clinical samples. (L)100 bp DNA ladder. Lane 1 to 12 is genomic DNA extracted from clinical samples

Ninety smear positive and negative clinical samples were subjected for genomic DNA extraction, then the DNA was extracted from 47 smear positive and 43 smear negative clinical samples of bone marrow (20) and spleen (70) aspirates successfully (Fig. 1). The frequency of suspected VL (*L. donovani*) patients based on sex and age groups is shown in (Table 1). Of 90 specimens, 47 (52.2%) samples were positive and 43 (23.8%) samples were negative by microscopic method (Table 2). By ITS1-PCR method, 48 (53.3%) clinical samples were confirmed as *L. donovani* positive while 42 (46.7%) were negative.

**Table 1:** Frequency of suspected patients to *L.donovani* based on sex and age groups

Variable	Characteristics	Frequency	Percent (%)
Sex	Male	87	96.7
	Female	3	3.3
Age	≤ 19	14	15.6
	20–39	58	64.4
	40–59	16	17.8
	≥ 60	2	2.2
	Total	90	100

**Table 2:** Frequency of positive to *Leishmania donovani* based on diagnostic techniques

Assay	Frequency (%)	
	Positive	Negative
Microscopic test	47 (52.2%)	43 (47.8%)
ITS1-PCR test	48 (53.3%)	42 (46.7%)

### PCR amplification of ITS1 region

Serial dilutions of *L. donovani* (*Sidon/LGR/2*) reference strain DNA (100 ng/µl) led to different detection thresholds of ITS1-PCR method; Tenfold dilution was prepared with 100 ng to 1 fg DNA concentration. Agarose gel electrophoresis (2%) of ITS1-PCR (320 bp) were amplified by LITSR/L5.8S primer at the concentration of (100 ng, 10 ng, 1 ng and 0.1 ng) DNA. But the DNA concentration ≤ 10^−2^ ng/µl was not detectable by this method.

**Fig. 2:**
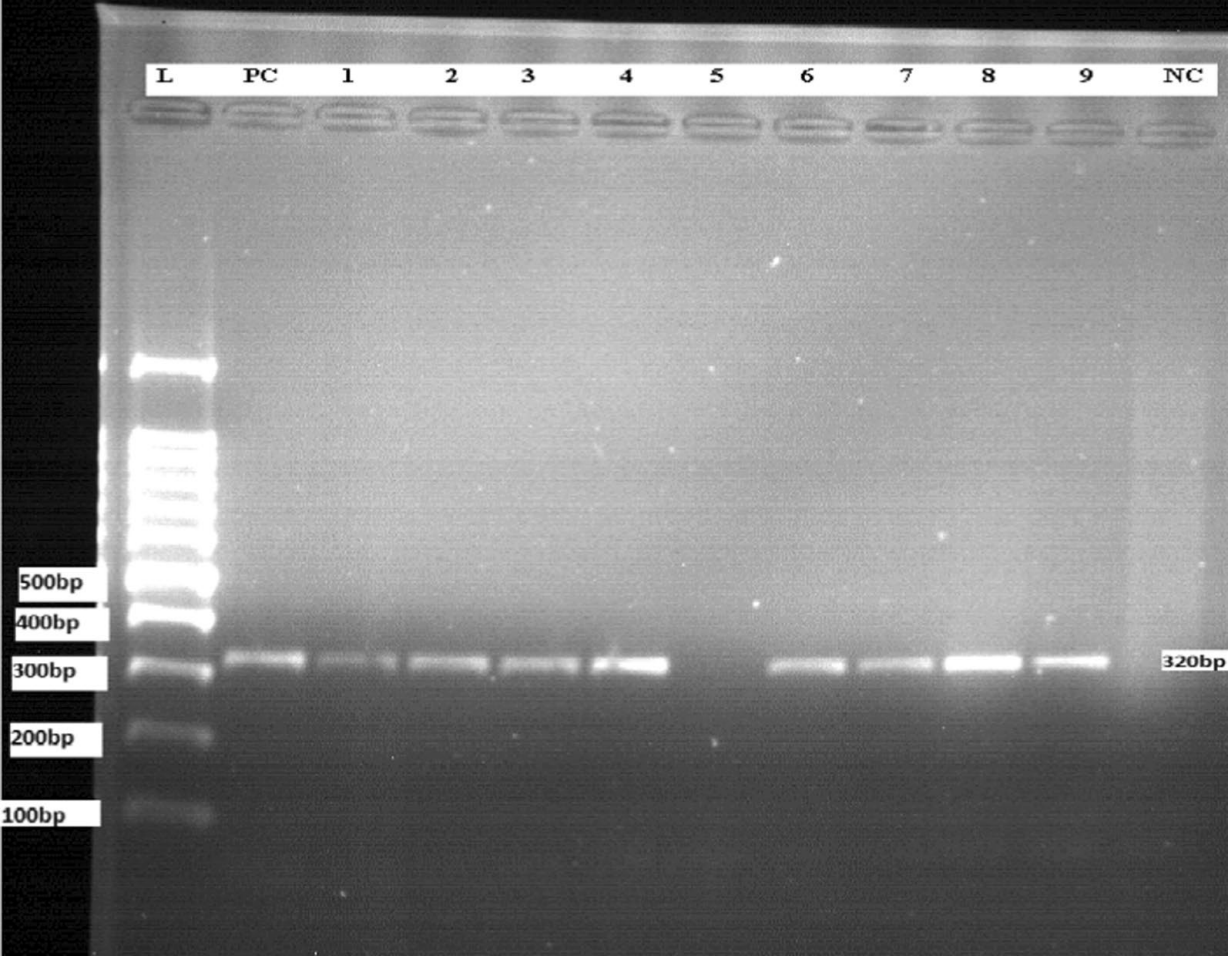
A representative agarose gel electrophoresis (2%) of ITS1-PCR (320 bp) from positive smear clinical samples. (L) 100 bp DNA ladder. (PC) *L. donovani* positive control DNA. (Nc) Negative control PCR master mix without DNA. Lane 1, 2, 3, 4, 6, 7, 8, 9 were positive to *L.donovani* while lane 5 was negative

The ITS1-PCR was performed on a total of 90 smears negative and smears positive samples. Agarose gel electrophoresis (2%) was done on ITS1-PCR amplicon using LITSR/L5.8S primers, the results showed that 48 total positive from both smear positive and negative clinical samples; which is 42/47 (89.4%) and 6/43 (13.9%) respectively (Fig. 2 and Additional files 1, 2: Figure S1, Figure S2).

### PCR–RFLP analysis of ITS1 region

**Fig. 3:**
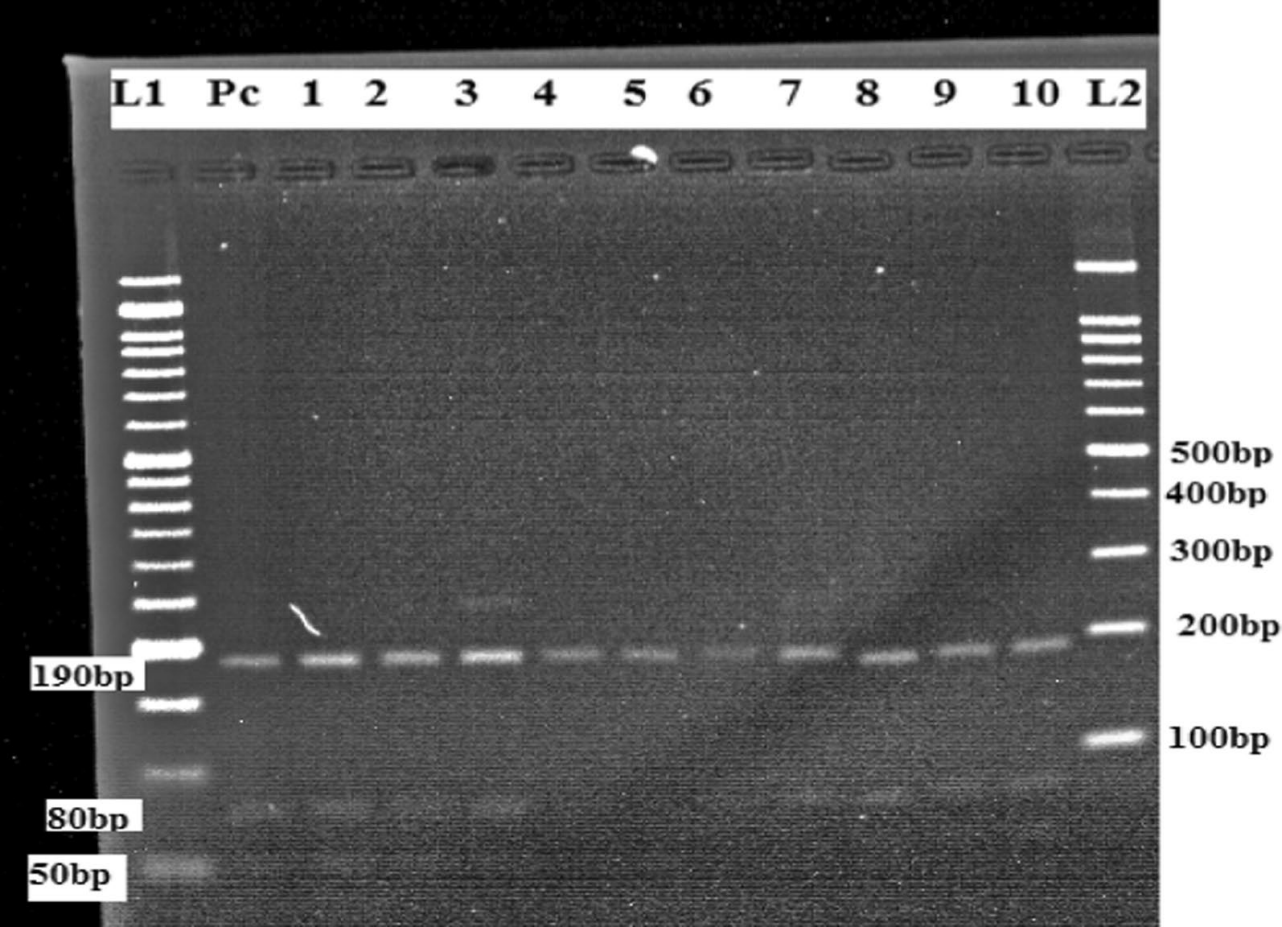
A representative agarose gel electrophoresis (2.5%) of PCR- RFLP method using *Hae* III restriction enzyme. (L1) 50 bp DNA ladder. (L2) 100 bp DNA ladder. (PC) positive control for *L.donovani* (190, 80 and 50 bp). Lane 1 to 10 was positive to *L. donovani* which provided (190, 80 and 50 bp) bands, Lane 3 contained extra band which indicates mixed co- infection

The ITS1-PCR products showed an amplified fragment of 320 bp for *L. donovani* species. All 48 ITS1-PCR positive samples were subjected to PCR- RFLP for confirmation of *Leishmania* species. After PCR- RFLP (*Hae* III) analysis of all positive clinical samples, its subsequent digestion revealed three different bands (50, 80, and 190 bp), corresponding to the pattern of *L. donovani* positive control. Moreover, in this study extra bands were detected, suggesting that there is a co-infection (Fig. 3) (Additional file 3).

### The efficiency of PCR- RFLP method for *Leishmania donovani*

The ITS1-PCR method provided 42 (89.4%) true positive, 6 (14.0%) false positive, 37 (86.0%) true negative, and 5 (10.6%) false negative with a total number of 48 (53.3%) positive and 42 (46.7%) negative results. The sensitivity, specificity, positive predictive value **(**PPV) and negative predictive value **(**NPV) of this specific study was 89.4%, 86.0%, 87.5%, and 88.1%, respectively (Table 3 and Additional file 3: Table S3).

**Table 3:** The efficiency of ITS1 PCR- RFLP Diagnostic Method for *Leishmania donovani* species considering Microscopy as the gold standard at 95% confidence interval (CI)

Method	Gold-standard Microscope		Missed value	Total	PPV	NPV	Kappa value	P-value
	Positive	Negative						
	(= 47)	(= 43)	0	(= 90)				
ITS1-PCR (LITSR/L5.8S)								
Positive	42(87.5%)	5 (11.9%)	0	47	89.4%	86.04%	0.755 (0.60–0.80)	P < 0.05
Negative	6 (12.5%)	37 (88.1%)	0	43				

### The association between Microscope and ITS1 PCR- RFLP results

The results of the current study indicated that there was a statistically significant association between Microscope and ITS1 PCR- RFLP test, since the goodness of fit χ^2^-value (51.305) greater than the table value (3.841) or P-value (0.000) < 0.05 (Table 3).

### The measures of agreement between Microscope and ITS1 PCR- RFLP results using kappa value

The measures of agreement between the two methods were with a kappa value of 0.755 (75.5%) and Monte Carlo significance at 95% Confidence interval with lower bound 0.000 and upper bound 0.033 which is P-value < 0.05. Therefore, the k value is between 0.60 and 0.80 which represents a substantial level of agreement between the two methods (Table 3 and Additional file 3: Table S3).

## Discussion

Visceral leishmaniasis which causes *L. donovani* affects the rural poor community and usually outbreaks occur during harvesting seasons [31]. Visceral leishmaniasis remains a public health problem particularly in migrant and nonimmigrant laborers involved in agricultural activities in Northwest Ethiopia [31]. In this study the frequency of suspected patient to VL (*L.donovani*), male is 96.7% and female is 3.3% while the age groups between, 20 and 39 are 64.4%. The male whose age groups between 20 and 39 are the most affected one. A large numbers of highly productive laborers were migrated to wards this area, this is due to growing of mechanized agricultural activities, therefore VL cases have becoming increased.

The present study showed that the PCR amplifying the ITS1 region using (LITSR/ L5.8S) primer a sensitive and accurate method for detecting low parasite load and a simple approach of extracting DNA from Giemsa-stained archived smear slide samples. The lysis buffer used to moisten the smears of the slide is effective in decreasing airborne contamination during content removal and collection [22].

The size and nucleotide sequence of the ITS1 region that separates the ssu rRNA and 5.8S rRNA genes differed amongst *Leishmania* species. The main advantage of the ITS1- RFLP is identification of species which can be achieved by digesting the PCR product with the restriction enzyme *Hae*III. Thus, all clinically essential species groups can be distinguished by their RFLP patterns [28]. The present study conducted DNA extracted from 90 bone marrow and spleen aspirate smear positive (47) and smear negative (43) clinical samples for visceral leishmaniasis, which was reported by *Leishemania* Research and Treatment Center.

Based on the findings of the present research using the ITS1-PCR method, it is possible to detect DNA at the concentrations of 100 ng, 10 ng, 1 ng and 0.1 ng, which is in agreement with a comparative study conducted by Mouttaki et al. [5] and better than the study conducted by Mohammadiha et al. [32]. Several previous studies were conducted to determine the detection limit of ITS1-PCR method, which are better than the results of present study [15, 19, 33]. This might be due to different types of PCR and the primer set used, different detection limits were reported by researchers.

Different bone marrow and spleen aspirate smear samples were amplified by ITS1- PCR using LITSR/L5.8S primer**,** the amplified PCR products from all positive clinical samples exhibiting moderate to strong ITS1 (320 bp) bands corresponding to the DNA of *L. donovani (Sidon/LGR/2)* reference strain, this study is in line with the study reported by Yaseen and Ali [34] and different to Hitakarun et al. [33] who conducts ITS1-PCR method to differentiate *L. siamensis* from *L. donovani* using a single-tube PCR, amplified at 348 bp and 319 bp respectively. Forty two (89.4%) and 6 (13.95%) clinical slide samples were detected as positive from 47 microscopic positive and 43 negative slide samples respectively, whereas 5/47 (10.6%) and 37/43 (86.05%) were detected as a negative by ITS1-PCR method. The present study detected true positive, false positive, true negative and false negative results from microscopically reported VL (*L.donovani*), this result is consistent with previous study reported by Hitakarun et al. [33] on comparison of PCR methods for detection of *Leishmania siamensis* infection, i.e., the cpb, the cyt b, the hsp70, the spliced leader mini-exon, the ssu-rRNA, the tim and the ITS1-PCR methods, upon amplifying the results of the PCR and their sequencing, all methods have produced false positive results except ITS1-PCR method. This indicates that ITS1-PCR method is highly specific compared to other methods. Some of microscopically reported clinical sample differed from ITS1-PCR method; this might be due to patient suffered from another co-endemic illness presenting similar clinical symptoms such as tuberculosis, enteric fevers, malaria or hepatitis and due to high specificity of the current method [35].

The sensitivity of the ITS1 analysis has been demonstrated and PCR–RFLP with *Hae*III is an efficient technique for the identification of *Leishmania* species (24). The current study conducted PCR–RFLP to determine clinical samples at species level. The PCR products of all positive clinical samples were subjected to PCR–RFLP analysis along with the DNA of *L.donovani (Sidon/LGR/2)* reference strains. *Leishmania* species were identified as belonging to the *L. donovani* species by PCR–RFLP patterns and the digestion revealed three different bands (190 bp, 80 bp and 50 bp), this study is similar to Kumar et al. [29]. Other similar studies were performed and the digestion provides three banding patterns; L*. infantum* (200, 100, and 50 bp) (5), *L*.*donovani* complex (184, 75, 54 bp) (26) and *L.donovani* (187, 75, 54) [19]. The results of the current study able to detect extra bands, suggesting a coinfection in few clinical samples along with the DNA of *L. donovani* reference strains, lane 3 of Fig. 3; this result was in agreement with a previous study in ITS1-RFLP analysis to identify *L. mexicana* in clinical samples [36].

The parasitological methods are the gold standard for the determination of VL with a high dependence on the number of parasites in tests and require technical skills for examining. The sensitivity of these methods is variable from 27 to 85% for the diagnosis of leishmaniasis and it is the most drawback of this method. Meanwhile, parasitological methods are not able to identify the species of *Leishmania* isolates [30]. The advantages of PCR in the identification of *L. donovani* species complex have great potential as a valuable tool in the detection of parasite DNA. Several studies of visceral leishmaniasis, which is caused by *Leishmania* parasites, have compared the ITS1-PCR diagnosis with conventional method. ITS1-PCR based method can identify the species of parasite involved and showed more sensitivity than the parasitological methods [26]. Even though ITS1- PCR is highly sensitive and more specific, it is not easily affordable and cost effective like that of microscopic method. According to results of the current study and considering microscopy as the gold standard, the sensitivity, specificity, PPV and NPV of the ITS1 -RFLP technique is 89.4%, 86%, 87.5%, and 88.1%, respectively, this result is comparable to the research reported by Kumar et al. [29] the sensitivity and specificity of the assay was 82.75% and 100% respectively while the PPV and NPV were 100% and 37.5% respectively. In some groups of human clinical sample 52.5% sensitivities was obtained by de Godoy et al. [20] using ITS1- RFLP which is less compared to the results of present study. The sensitivities and specificity of present study was less than the results reported by Ranasinghe et al. [19].

The statistical analysis of the present study showed that there is a significant association between the microscopic result and ITS1- RFLP methods, since P-value is < 0.05. The study conducted by Hajjaran, et al. [30] reported that 51 (85%) of 60 microscopy-positive slides were also positive by the ITS1- RFLP assay; kappa analysis revealed that there was 61.8% agreement between microscopy and the ITS1-RFLP assay (P-value < 0.05) which is a relatively good concordance. In this study the strength of agreement between the ITS1 -RFLP and gold standard microscopy methods, a substantial level of agreement was observed, with a kappa value (0.741) between 0.6 and 0.80 which is 74.1% (Table 2) and P-value (0.00–0.03) < 0.05. The ITS1 -RFLP method was detected 48 out of 90 microscopic positive and negative clinical samples; this indicates that the current study was better than that of the previous study.

## Conclusion

Based on the current study, positive and negative results were obtained from smear negative and positive samples using ITS1- RFLP method. According to the results, the ITS1-PCR method amplifies the target region of DNA at the concentration starting from 0.1 µg/µl and above. All ITS1-PCR positive visceral leishmaniasis clinical samples belong to *L.donovani* species by ITS1- RFLP method except those mixed co-infections. Up on restriction of digestion, three different RFLP patterns were obtained which is similar to the *L. donovani* reference strain. Therefore, the ITS1- RFLP method is highly sensitive on smear negative samples, depending on statistical analysis of the present finding there is significant association and substantial levels of degree of agreement between gold standard microscopic and ITS1- RFLP method, since the P-value is < 0.05. Compared to a parasitological report by direct microscopy, ITS1- RFLP is a better way to diagnose *Leishmania donovani* species, especially for smear negative clinical samples.

### The limitation of the study

The major limitation of this study was it does not compare between different molecular diagnostic techniques, this is due to unable to import some primers because of limitation of funds and the occurrence of covid-19 pandemic disease in the world.

## Supplementary Information

**Additional file 1: Figure S1**. Agarose gel eclectrophoresis (2%) of ITSI-PCR (320pb) from positive smear clinical samples.

**Additional file 2: Figure S2**. Agarose gel eclectrophoresis (2%) of ITSI-PCR (320pb) from positive smear clinical samples.

**Additional file 3: Table S3**. Staatical analysis of PCR-ITS1 * microscopic results of Crosstabulation, Chi-Square Tests and Kappa values.

## Data Availability

Data supporting the conclusions of this article are included within the article and in the additional information.

## Abbreviations

CL: Cutaneous leishmaniasis
ITS: Internal transcribed spacers
kDNA: Kinetoplastid DNA
NASBA: Nucleic acid sequence-based amplification
RFLP: Restriction fragment length polymorphism
ssrRNA: Small subunit ribosomal RNA
VL: Visceral leishmaniasis
CPCR: Conventional single-step PCR
qPCR: Quantitative real-time PCR
NPCR: Nested PCR
hsp70: Heat shock protein 70
cpb: Cysteine protease B
cyt b: Cytochrome b
DNDi: Drugs for Neglected Diseases Initiative
