# Supplementary figures and images for: Detection of Leishmania donovani using ITS1-RFLP from positive and negative smear samples among clinically reported patients visiting University of Gondar Comprehensive Specialized Hospital
Source: BMC Infect Dis. 2022 Dec 29;22:963. doi: 10.1186/s12879-022-07930-1 (PMC9797380; doi:10.1186/s12879-022-07930-1)

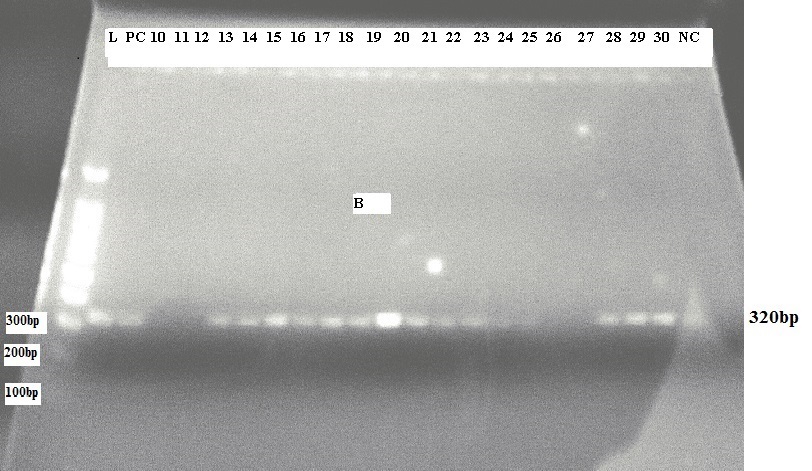

Supplement: Supplementary file 1 — Additional file 1: Figure S1. Agarose gel eclectrophoresis (2%) of ITSI-PCR (320pb) from positive smear clinical samples. [file 12879_2022_7930_MOESM1_ESM.jpg]

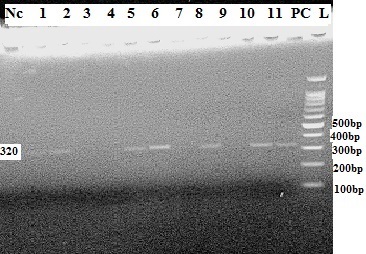

Supplement: Supplementary file 2 — Additional file 2: Figure S2. Agarose gel eclectrophoresis (2%) of ITSI-PCR (320pb) from positive smear clinical samples. [file 12879_2022_7930_MOESM2_ESM.jpg]
