# Supplementary material for: Detection of Leishmania donovani using ITS1-RFLP from positive and negative smear samples among clinically reported patients visiting University of Gondar Comprehensive Specialized Hospital
Source: BMC Infect Dis. 2022 Dec 29;22:963. doi: 10.1186/s12879-022-07930-1 (PMC9797380; doi:10.1186/s12879-022-07930-1)
Supplement: Supplementary file 3 — Additional file 3: Table S3. Staatical analysis of PCR-ITS1 * microscopic results of Crosstabulation, Chi-Square Tests and Kappa values. [file 12879_2022_7930_MOESM3_ESM.docx]

| **Case Processing Summary** | | | | | | |
| --- | --- | --- | --- | --- | --- | --- |
|  | Cases | | | | | |
|  | Valid | | Missing | | Total | |
|  | N | Percent | N | Percent | N | Percent |
| PCR-ITS1 Results * microscopic results | 90 | 100.0% | 0 | 0.0% | 90 | 100.0% |

| **PCR-ITS1 Results * microscopic results Crosstabulation** | | | | | |
| --- | --- | --- | --- | --- | --- |
|  | | | microscopic results | | Total |
|  |  |  | posetive | negative |  |
| PCR-ITS1 Results | posative | Count | 42 | 6 | 48 |
|  |  | % within microscopic results | 89.4% | 14.0% | 53.3% |
|  | negative | Count | 5 | 37 | 42 |
|  |  | % within microscopic results | 10.6% | 86.0% | 46.7% |
| Total | | Count | 47 | 43 | 90 |
|  |  | % within microscopic results | 100.0% | 100.0% | 100.0% |

| **Chi-Square Tests^c^** | | | | | | |
| --- | --- | --- | --- | --- | --- | --- |
|  | Value | df | Asymp. Sig. (2-sided) | Exact Sig. (2-sided) | Exact Sig. (1-sided) | Point Probability |
| Pearson Chi-Square | 51.305^a^ | 1 | .000 | .000 | .000 |  |
| Continuity Correction^b^ | 48.319 | 1 | .000 |  |  |  |
| Likelihood Ratio | 57.757 | 1 | .000 | .000 | .000 |  |
| Fisher's Exact Test |  |  |  | .000 | .000 |  |
| Linear-by-Linear Association | 50.734^d^ | 1 | .000 | .000 | .000 | .000 |
| N of Valid Cases | 90 |  |  |  |  |  |
| a. 0 cells (0.0%) have expected count less than 5. The minimum expected count is 20.07. | | | | | | |
| b. Computed only for a 2x2 table | | | | | | |
| c. For 2x2 crosstabulation, exact results are provided instead of Monte Carlo results. | | | | | | |
| d. The standardized statistic is 7.123. | | | | | | |

| **Symmetric Measures** | | | | | | | | |
| --- | --- | --- | --- | --- | --- | --- | --- | --- |
|  | | Value | Asymp. Std. Error^a^ | Approx. T^b^ | Approx. Sig. | Monte Carlo Sig. | | |
|  |  |  |  |  |  | Sig. | 95% Confidence Interval | |
|  |  |  |  |  |  |  | Lower Bound | Upper Bound |
| Measure of Agreement | Kappa | .755 | .069 | 7.163 | .000 | .000^c^ | .000 | .033 |
| N of Valid Cases | | 90 |  |  |  |  |  |  |
| a. Not assuming the null hypothesis. | | | | | | | | |
| b. Using the asymptotic standard error assuming the null hypothesis. | | | | | | | | |
| c. Based on 90 sampled tables with starting seed 2000000. | | | | | | | | |
